# Optimization-Based Controllers for Robotics Applications (OCRA): The Case of iCub’s Whole-Body Control

**DOI:** 10.3389/frobt.2018.00024

**Published:** 2018-03-29

**Authors:** Jorhabib G. Eljaik, Ryan Lober, Antoine Hoarau, Vincent Padois

**Affiliations:** Sorbonne Université, CNRS UMR 7222, Institut des Systèmes Intelligents et de Robotique, ISIR, Paris, France

**Keywords:** whole-body controller, iCub, optimization, tasks, hierarchical, code:c++

## Abstract

OCRA stands for Optimization-based Control for Robotics Applications. It consists of a set of platform-independent libraries which facilitates the development of optimization-based controllers for articulated robots. Hierarchical, weighted, and hybrid control strategies can easily be implemented using these tools. The generic interfaces provided by OCRA allow different robots to use the exact same controllers. OCRA also allows users to specify high-level objectives via tasks. These tasks provide an intuitive way of generating complex behaviors and can be specified in XML format. To illustrate the use of OCRA, an implementation of interest to this research topic for the humanoid robot iCub is presented. OCRA stands for Optimization-based Control for Robotics Applications. It consists of a set of platform-independent libraries which facilitates the development of optimization-based controllers for articulated robots. Hierarchical, weighted, and hybrid control strategies can easily be implemented using these tools. The generic interfaces provided by OCRA allow different robots to use the exact same controllers. OCRA also allows users to specify high-level objectives via tasks. These tasks provide an intuitive way of generating complex behaviors and can be specified in XML format. To illustrate the use of OCRA, an implementation of interest to this research topic for the humanoid robot iCub is presented.

## 1 Introduction

Whole-body control (WBC) is a research direction in robotics, where humanoids are faced with the problem of executing multiple tasks simultaneously. As stated by the IEEE Technical Committee on Whole-Body Control:

A control system that is specifically designed to guarantee the execution of a single task, even if it uses all the joints of a robot, cannot be considered WBC.

This is indeed the core of the software introduced in this work, but it goes further by drawing additional requirements from the identification of typical concerns in the control of articulated robots, such as (1) standardization of the problem formulation, which is done in the form of an optimization problem; (2) flexibility in the solver choice; (3) independence of tasks from the problem formulation with user-friendly ways to introduce them; (4) addition of constraints, contact modeling and support for both fixed and floating-base robots. OCRA draws its origins from these design requirements. It stands for Optimization-based Control for Robotics Applications and consists of a set of platform-independent libraries which facilitates the development of optimization-based controllers. It builds on top of ORC which was originally a framework developed by CEA-List,^1^ later used at the Institute of Intelligent Systems and Robotics (ISIR) to develop whole-body controllers with simulations on XDE (Salini et al., 2013).

Examples of software addressing similar problems include the Stack of Tasks (SOT) (Mansard et al., 2009), OpenSOT (Rocchi et al., 2015), and CoDyCo^2^ controllers (Nori et al., 2015). Nevertheless, they either lack the level of desired flexibility or do not meet the proposed design requirements. SOT and OpenSOT use strictly hiearchical methods, and while OpenSOT is intended for torque-controlled robots similar to OCRA, SOT originally targets velocity-controlled robots. When it comes to solvers, OpenSOT relies solely on QPOases while SOT’s controller and solver are tight together.

Another software that has been used in the formulation of this type of controllers is Roboptim (2016). It is, however, an optimization framework for robotics and it is up to the user to formulate the control problem, workout the prioritization strategy and address the different components to achieve a whole-body controller.

CoDyCo’s controllers on the other hand, although aimed at WBC, are tailored to be task-specific and do not constitute a WBC library.

OCRA has been designed to exploit a client–server paradigm, where the *server* is responsible for running the whole-body controller, send control inputs to the robot and host user-defined tasks, while the *client* is built by the user according to their needs on task servoing, planning, or higher-level control.

OCRA contributes to the building of the iCub mindware through the implementation of an *iCub server* along with communication utilities for the construction of clients. It facilitates the creation of a vast type of whole-body behaviors, with special attention to interaction. It also addresses the needs of different types of users, from the advanced one who desires to implement particular low-level control laws, to the more practical one who prefers to state at the metatask-level.

In Section 2, a generic overview of the main design requirements and features of OCRA, along with a list of software dependencies is presented. Section 3 introduces the main concepts involved in optimization-based control which allow the reader to have a deeper insight in the inner workings of the software. Concepts such as tasks, constraints, quadratic programming based control (and motivations for its use), prioritization strategies, and optimization solver are covered. Section 4 spans OCRA’s structure, shedding light on its libraries and the main classes they are composed of as well as how these were used for iCub implementations. The same section continues with a more in-depth description of the iCub server and a generic client through sequence diagrams, as well as a brief explanation on how to automatically build a template client. Finally, Section 5 draws final conclusions.

## 2 OCRA

OCRA is a set of libraries and tools for the implementation of QP-based whole-body controllers for torque/force-controlled articulated robots. Robots like the humanoid iCub or the KUKA Light Weight Robot (LWR) manipulators (floating/fixed base) can be controlled using this open source software. In particular, for the iCub, the set of necessary libraries is implemented and distributed.

One main design requirement from OCRA’s inception is that (1) it should be heavily task-oriented. This means, that a user can specify a set of tasks to be performed by the robot, e.g., *follow a CoM trajectory, while maintaining balance and make one hand follow another trajectory* and (2) the specifications of these tasks have to be easy to provide. This is achieved through an XML file that we call the *tasks set*.

Features that make OCRA flexible include: the possibility to choose between different types of tasks and their prioritization strategies; two different optimization solvers; various types of constraints and the tools to create a client–server architecture, where the *server* runs a reactive controller with the tasks and constraints, and one or more *clients* perform the computation of the right instantaneous tasks values through local trajectory controllers (e.g., PIDs), motion planning, model predictive control, or any higher-level control schemes.

The required dependencies of this software are given in Table 1.

**Table 1:** Required dependencies table for ocra and ocra-icub.

*Dependency*	*Minimum version*	*ocra*	*ocra-icub*
YARP	2.3	✓	✓
Eigen	3.2	✓	✓
orocos_kdl	1.2	✓	✓
iDynTree	0.4.0		✓
yarpWholeBodyInterface	0.35		✓
Boost	1.64	✓	✓
CMake	2.8.11	✓	✓
TinyXML	2.6.2	✓	
YCM	0.4.0		✓

*For the sake of clarity, it is not shown that ocra is naturally a dependency of ocra-icub*.

## 3 Optimization-Based Control

Traditionally, redundancy resolutions for robotic control problems find analytical solutions by ensuring that lower-priority tasks are executed in the null-space of higher-priority tasks. In prioritized inverse kinematics, acceleration or torque based control, the jacobian of low-priority tasks is projected onto the null-space of higher-priority ones (Khatib, 1987; Sentis and Khatib, 2006; Peters et al., 2008). Inequality constraints are, however, difficult to deal with in these approaches. They are usually transformed into avoidance tasks, which try to prevent the robot from hitting the original constraint (Khatib, 1986; Padois et al., 2007). This type of active avoidance (passive or active) method is doomed to fail as the number of constraints is necessarily higher than the number of DOF (2*n* joints limits for an *n* DOF robot) and it thus requires to make decision reactively about which avoidance tasks should be used in order to guarantee the respect of all constraints while still achieving the operational tasks in the most efficient way possible (Padois, 2016).

OCRA resorts to convex optimization for the formulation of the whole-body controller, as it has been stated multiple times before this point. The controller is written as a linearly constrained quadratic multi-objective optimization problem where strict or soft hierarchies are used to express the priorities between the tasks. *Linearly constrained* due to the constraints being strictly linear (or linearized if not), *quadratic* because each objective is the quadratic error of a task and *multi-objective* because multiple tasks are combined. The result of this optimization are the optimal actuation inputs to the system (i.e., joint torques) given the set of prioritized tasks to be performed and the constraints that have to be respected. Among these constraint, this optimization problem includes inequality constraints, coming from control input saturations or any other variable which should never cross certain limits. Under these conditions, the solution space can be proved convex and finding the optimal solution to the whole-body control problem is equivalent to finding the set of active constraints. In fact, methods in which optimization is avoided end up using algorithms that pretty much search for this active set, not explicitly and in a suboptimal way. It is then indisputable that the strong background in convex optimization outruns analytical methods used to heuristically activate constraints.

The primary concern of this section is to present the necessary equations and relationships to understand the critical aspects of the types of controllers which can be developed with OCRA. Generally speaking, an optimization-based controller formulates the control problem as one of minimizing control objective functions while respecting the control constraints. Specifically, the problem is formulated as a convex linearly constrained QP using the second-order rigid body dynamics of the robot. Therefore, the control objectives (Tasks) are expressed as either accelerations, torques, or wrenches, allowing for complex dynamic interactions with the environment, and the control constraints are expressed directly in the QP as linear equalities and inequalities.

### 3.1 Tasks

Tasks allow users to decompose complex whole-body behaviors into atomic control objectives, which can be planned by a user or automatically with planners. Here, a task represents a control objective for the robot, and more specifically, an error between some desired task value and the current value of the task in terms of the control variable. These tasks are expressed as the squared norm of these errors in either accelerations, torques, or wrenches and can be expressed in both joint and operational-space. In Section 3.4, the expression of these tasks in terms of the control variables is provided, but Table 2, below, shows their standard formulations.

**Table 2:** Different types of tasks.

Task	Definition
Operational-space acceleration	$\text{T}\;\left({\ddot{\xi }}^{\text{des}}\right)\;=∥\text{J}\left(\text{q}\right)\dot{\nu }+\dot{\text{J}}\left(\text{q},\nu \right)\nu -{\ddot{\xi }}^{\text{des}}∥$
Joint-space acceleration	$\text{T}\;\left({\dot{\nu }}^{\text{des}}\right)\;=∥\dot{\nu }-{\dot{\nu }}^{\text{des}}∥$
Operational-space wrench	$T{(}^{e}\omega^{\text{des}})=\left\|{}^{e}\omega {-}^{e}\omega^{\text{des}}\right\|$
Joint torque	$\text{T}\;\left(\tau^{\text{des}}\right)\;=∥\tau -\tau^{\text{des}}∥$

*Superscript “des” stands for desired*.

In Table 2, ***ν*** and $\dot{\nu }$ are the generalized velocities and accelerations of the robot. They can be more or less directly related to the derivatives of the generalized coordinates ***q***. Indeed, for robots whose root link can float freely in Cartesian space, e.g., humanoids, it is necessary to consider the pose of the root link w.r.t. the world reference frame. The primary method for doing so is to account for the root link pose directly in the generalized coordinates, ***q***, of the robot (Sentis and Khatib, 2005; Mistry et al., 2010). The terms *J* and $\dot{J}$ are link Jacobians and their derivatives. The variable *^e^****ω*** represents an external wrench, and ***τ***, the system torques, while $\ddot{\xi }$ is operational-space acceleration. The corresponding *desired* values of each term in Table 2 should not be confused with the raw trajectory given by the user (subscript *ref*). These set-points are used as inputs to a task-level PD controller in the case of operational-space acceleration tasks and a PI in the case of wrench (*^e^****ω***) tasks, such that:

(1) $$\begin{array}{l} {\ddot{\xi }}^{\text{des}}\left(t+\Delta t\right)={\ddot{\xi }}^{\text{ref}}\left(t+\Delta t\right)+K_{p}\epsilon \left(t\right)+K_{d}\dot{\epsilon }\left(t\right), \end{array}$$

(2) $$\begin{array}{l} {\;}^{e}\omega^{\text{des}}\left(t+\Delta t\right){=}^{e}\omega^{\text{ref}}\left(t+\Delta t\right)+K_{p}\epsilon \left(t\right)+K_{i}\int \epsilon \left(t\right)dt, \end{array}$$

where ${\ddot{\xi }}^{\text{ref}}$ and *^e^****ω***^ref^ are feedforward terms, while ***ϵ*** and $\dot{\epsilon }$ are pose error and its derivative (these being representation dependant). *K_p_*, *K_d_*, and *K_i_* are proportional, derivative, and integral gains and by default, $K_{d}=2\sqrt{K_{p}}$. Task servoing is necessary to compensate for drift and tracking errors associated with using second-order control techniques. Additionally, it is often the case that only position values are specified by the user, and these must be converted to accelerations—task servoing provides this service. For joint-space accelerations the servoing is done in similar fashion as for ${\ddot{\xi }}^{\text{des}}$.

### 3.2 Constraints

As with all real world control problems, there are limits to what the system being controlled can do. For example, the control input is typically bounded, which for robots with revolute joints means that the torque which can be generated by the actuators is limited to plus or minus some value. Likewise, the joints themselves generally have limited operating ranges for various mechanical reasons. In addition to these common limiting factors, it may be reasonable to maintain the robot in some region of its state space that will ease control, e.g., avoid slipage of the contact points or avoid contact with the environment.

In Table 3, the •_min_ and •_max_ values represent the lower and upper limits of a variable. The term ${C_{c_{j}}}^{e}\omega_{j}\leq 0$ represents the linearized friction cone constraint for a point contact, and ${\;}^{e}J\left(q\right)\dot{\nu }{+}^{e}\dot{J}\left(q,\nu \right)\nu =0$, its coupled “no motion” constraint, which ensures that the contact does not move. For details on these constraint expressions and the way to express them through linearization as functions of joint torques or generalized acceleration, the reader is directed to Salini et al. (2011). In addition to these nearly universal robotic constraints, particular care must be taken to ensure that the motions generated by the controller respect the system dynamics, i.e., the equations of motion.

**Table 3:** Possible constraints in OCRA.

*General constraint*	*Equation*
Actuator limits	***τ***_min_ ≤ ***τ*** ≤ ***τ***_max_
Joint position limits	***q***_min_ ≤ ***q*** ≤ ***q***_max_
Joint velocity limits	${\dot{\nu }}_{\text{min}}\leq \dot{\nu }\leq {\dot{\nu }}_{\text{max}}$
Contact constraints	${{\text{C}}_{{\text{c}}_{\text{j}}}}^{\text{e}}\omega_{\text{j}}\leq \text{0}$
	${\;}^{\text{e}}\text{J}\left(\text{q}\right)\dot{\nu }{+}^{\text{e}}\dot{J}\left(\text{q},\nu \right)\nu =\text{0}$

### 3.3 Dynamics

The principle constraint of the controllers in OCRA is that of the system dynamics. This means that any solution found must be dynamically feasible, and consequently, respect the equations of motion,

(3) $$M(q)\dot{\nu }+\underset{n(q,\nu )}{\underset{︸}{C(q,\nu )\nu +g(q)}}=S^{⊤}\tau {+}^{e}J^{⊤}{\left(q\right)}^{e}\omega$$

(4) $$\begin{array}{l} M\left(\text{q}\right)\dot{\nu }+\text{n}\left(\text{q},\nu \right)=S^{⊤}\tau {+}^{e}J^{⊤}{\left(\text{q}\right)}^{e}\omega . \end{array}$$

In (3), *M*(***q***) is the generalized mass matrix, *C*(***q***, ***ν***)***ν*** and ***g***(***q***) are the Coriolis-centrifugal and gravitational terms, *S* is a selection matrix indicating the actuated degrees of freedom, *^e^****ω*** is the concatenation of the external contact wrenches, and *^e^J* their concatenated Jacobians. Grouping *C*(***q***, ***ν***)***ν*** and ***g***(***q***) together into ***n***(***q***, ***ν***), we can simplify the equations to (4). Additionally, the variables $\dot{\nu }$, τ, and *^e^****ω***, can be grouped into the same vector,

(5) $$x=\left[\begin{array}{c} \dot{\nu }\\ \tau \\ {}^{e}\omega , \end{array}\right]$$

forming the *control variable*, and allowing (4) to be rewritten as,

(6) $$\underset{A}{\underset{︸}{\left[-M(q)\;S^{⊤}\;{}^{e}\;J^{⊤}(q)\right]}}x=\underset{b}{\underset{︸}{n(q,\nu )}}.$$

Equation 6 provides an affine equality constraint, *A****x*** = ***b***, which can be used to ensure that the minimization of the control objectives respects the system dynamics.

### 3.4 Quadratic Programming Based Control

Given the control objectives defined by the task errors from Section 3.1, the control constraints from Section 3.2, and the optimization variable defined by (5), we can now form a generic, single task, optimization-based whole-body control problem as,

(7) $$\begin{array}{ll} \underset{\text{x}}{\text{min}} & T_{i}\left(\text{x}\right)\\ \text{s.t.} & G\text{x}\leq \text{h}\\ & A\text{x}=\text{b}, \end{array}$$

where the objective function, *T_i_*(***x***), is the task error, representing for example, the squared error between a desired acceleration or wrench and the system’s (see Section 3.1). The inequality constraints, generically represented by, *G****x*** ≤ ***h***, contain the concatenation of all of the affine inequalities defined in Table 3, while the affine equality constraints, shown by *A****x*** = ***b***, obligatorily contain the equation of motion constraints from (6), and possibly the coupled “no motion” constraints of any contacts which might be active.

The form of this problem will be referred to throughout this work as the *full problem*, which is also the default formulation used in OCRA. The user can choose to work with the *reduced problem*, in which the dynamics are not explicit in the constraints, but projected onto the different control objectives, and with the optimization variable, ***x***, in this case, consisting of the control inputs, ***τ***, and external wrenches *^e^****ω***, i.e., $\text{x}={\left[\tau^{⊤}{\;}^{e}\omega^{⊤}\right]}^{⊤}$. The reduced problem has the advantage of having less optimization variables, which can improve the solution time as shown in Section 3.5 of Salini (2012), at the expense of complicating the writing of the tasks and constraints in terms of the optimization variable. The inclusion of the generalized joint accelerations, $\dot{\nu }$, in the full problem, yields clarity and simplicity when writing the cost functions and the constraints on the joint velocities, acceleration and joint limits.

### 3.5 Prioritization Strategies

Up to this point, only one task objective function is considered in the whole-body controller in Section 3.4. If multiple task objective functions are combined (using operations that preserve convexity) in the resolution of the control problem, then they can be performed simultaneously. In these cases, it is important to select a strategy for the resolution of the optimization problem. The strategy will in turn, determine how tasks interact/interfere with one another. The two prevailing methods for dealing with multiple tasks are hierarchical (Saab et al., 2013; Escande et al., 2014) implemented as WOCRA and weighted prioritization (Bouyarmane and Kheddar, 2011; Salini et al., 2011) implemented as HOCRA. A hybrid scheme can also be used providing the best of the former two methods (Liu et al., 2016).

## 4 Software

### 4.1 Structure

#### 4.1.1 OCRA Libraries

The main concepts introduced in previous sections are materialized in the different interfaces, abstract, and concrete classes OCRA is composed of. These are encapsulated in four essential components or libraries. These are: ocra-optim, ocra-control, ocra-coms, and ocra-utils.

The first of these libraries, ocra-optim, defines the lowest-level data structures required to build an optimization problem such as variables, functions, and constraints, as well as the basic concept of a solver and prioritization strategies. Table 4 shows the main classes in this library, their type, and a brief description.

**Table 4:** Main classes composing the ocra-optim library.

	ocra-optim

Main classes	Features
Variable 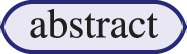	Represents the mathematical concept of variable
Function 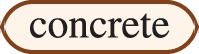	Base for any type of function
Constraint 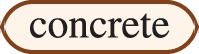	Templated base class to build equality/inequalities constraints
LinearizedCoulombFunction 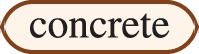	Builds a discretized cone representing a Coulomb Friction cone
Solver 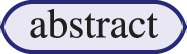	Base class for optimization solvers
CascadeQPSolver 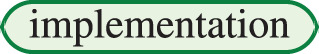	Implements a hierarchical solver
OneLevelSolver 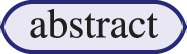	Used for building solvers with one level of importannce to all tasks. It also contains specific implementations with QuadProg++ and QPOases. This is the solver used in wocra

*Blue labels indicate abstract classes that can be later implemented. Orange labels are assigned instead to concrete classes without particular inheritances, while green labels stand for implemented classes*.

The ocra-control library goes up one level of abstraction, containing all the classes necessary to build the model of a robot, implement a control law, account for the floating-base dynamics and build the different types of tasks, constraints and trajectories. The two main prioritization techniques described in Section 3.5 are, respectively, implemented through HOCRA and WOCRA. Again, the main classes in this library along with their brief description are collected in Table 5.

**Table 5:** Main classes composing the ocra-control library.

ocra-control	
Main classes	Features
Controller 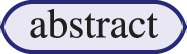	Used to implement control laws
Model 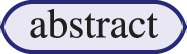	Provides dynamic and static terms from the equations of motion
FullDynamicEquationFunction 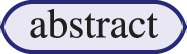	Creates the dynamics equation as a linear function of the optimization variable
ModelContacts 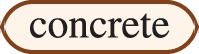	Concatenates the contact variables and Jacobians for a model
ControlFrame 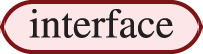	Generic representation of a frame
Feature 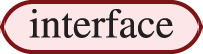	Used by tasks to compute errors and Jacobians
Task 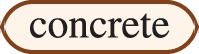	–
TaskBuilder 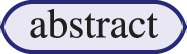	Builds task-specific features
*TaskBuilder 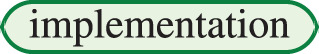	Task-specific implementations of TaskBuilder. “*” is replaced by Com, FullPosture, Orientation, etc.
TaskConstructionManager 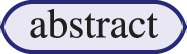	–
*LimitConstraint 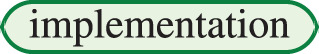	(torque and joint limits)
Trajectory 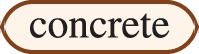	Helper class to build trajectories. These can be minimum jerk, linearly interpolated, gaussian processes or time-optimal
WocraController 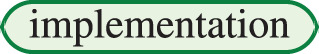	QP-based controller using a weighted prioritization strategy
HocraController 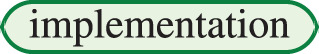	QP-based controller using a hierarchical prioritization strategy

*Blue labels indicate abstract classes that can be later implemented. Orange labels are assigned instead to concrete classes without particular inheritances. Red labels to interfaces and green labels to implementations*.

The last two libraries are agnostic to the paradigm suggested by OCRA. That is, a client–server model. In order to implement it, the ocra-coms library is provided and comes with the generic classes to create a server and a client and to manage the communication between them. Table 6 lists the main classes in this library along with their description.

**Table 6:** Main classes composing the ocra-coms library.

ocra-coms	
Main classes	Features
ControllerServer 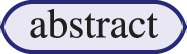	Must be inherited to implement the server side
ServerCommunications 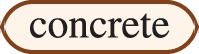	Helps the server establish YARP-based communication with the client
ClientCommunications 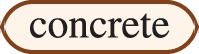	Helps the client establish YARP-based communication with the server
ClientManager 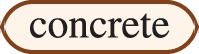	Implements the functionalities of YARP RFModule on the client side. Holds the main client thread
ControllerClient 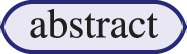	Implements the functionalities of YARP RateThread on the client side. Main thread hosted by ClientManager
TaskConnection 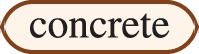	Used on the client side to connect and communicate with the tasks started by the server
TrajectoryThread 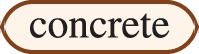	Used to create trajectories on the client side

*Blue labels indicate abstract classes that can be later implemented. Orange labels are assigned instead to concrete classes without particular inheritances*.

Finally, the ocra-utils library as its name states, is a set of utilities to aid the other libraries: helpers to perform file operations, xml parsing, data structure conversions, errors descriptors, among others.

#### 4.1.2 OCRA for iCub

The classes needed to *implement* a server for the iCub robot and a generic client are present in the ocra-icub library. As can be seen from the green implementation labels in Table 7, most of the main classes are implementations of base classes from ocra-control and ocra-coms. In the following section, two main detailed explanations are provided: how to use these classes to obtain a client–server architecture for iCub, and how objects of the different classes interact.

**Table 7:** Main classes composing the ocra-icub library.

ocra-icub	
Main classes	Features
ModelInitializer 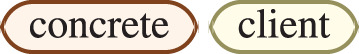	Retrieves model configuration information from the server to create a local copy of the robot model
OcraWbiModel 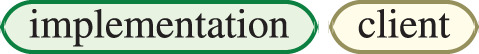	Implements the abstract Model class from ocra-control for the iCub robot
IcubControllerServer 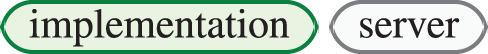	Implements ControllerServer for the iCub robot
Module 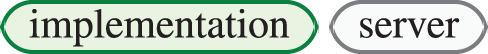	Module that launches the controller thread, parses controller options and the tasks set XML. Basically a yarp:os:RFModule
Thread 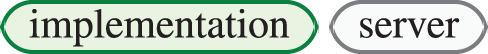	Main controller thread started. Created by Module, contains the controller, tasks manager, and solves the whole-body control problem

*Orange labels mean concrete class without any particular inheritance. Green labels are for classes that implement some base class from the main OCRA libraries. Yellow labels stand indicate classes that are used to build a client, while gray labels are for those used to build a server*.

**Figure 1:**
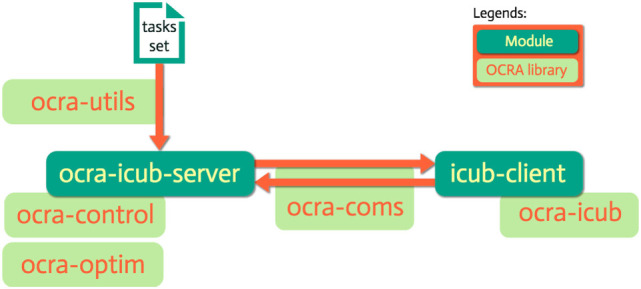
A server (ocra-icub-server) and a client (icub-client) are here represented in dark green as YARP modules. In light green, we see the underlying OCRA libraries associated to their construction, as well as for the communication between them and the parsing of the tasks set.

**Figure 2:**
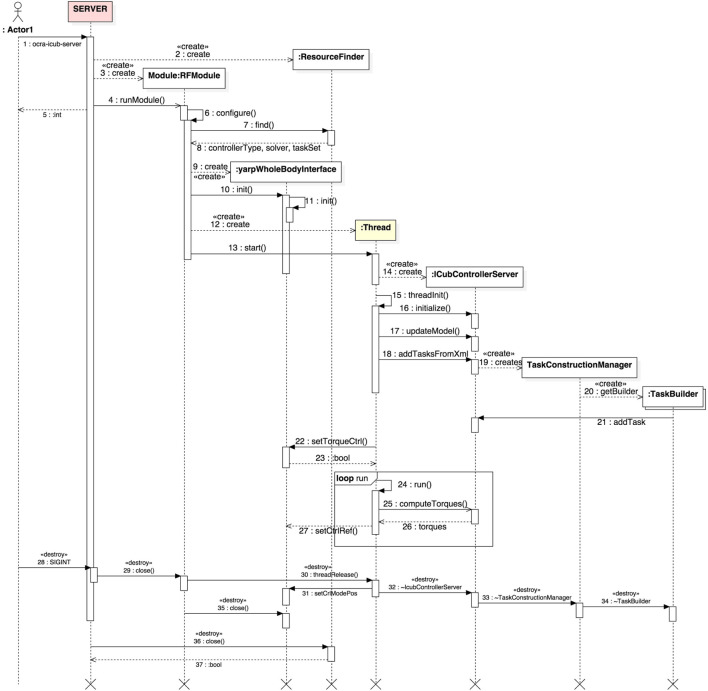
UML sequence diagram displaying the typical interactions within the ocra-icub-server. The time evolution of interactions is followed from top to bottom, while messages passed among objects are found in the horizontal dimension. The light yellow background of some lifelines indicates that these are threads.

**Figure 3:**
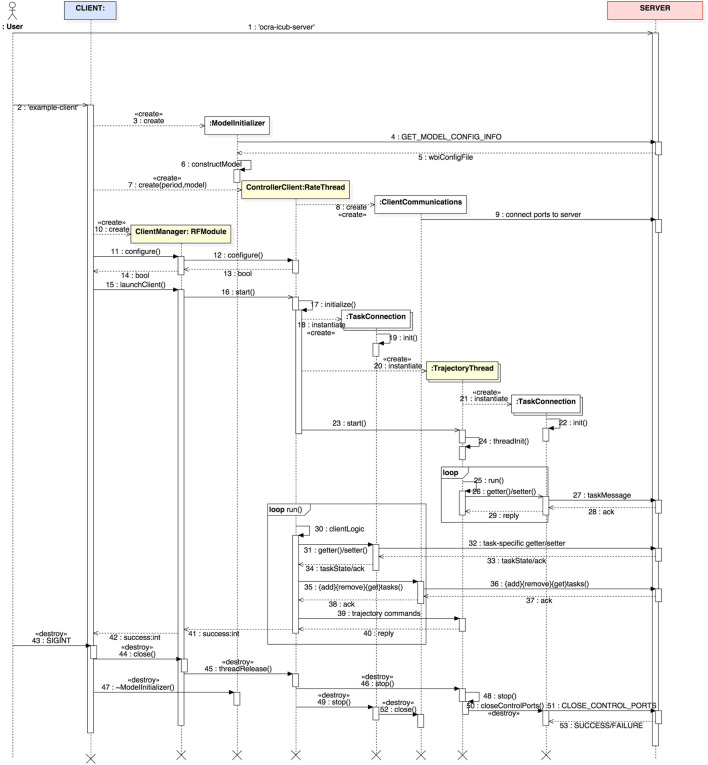
UML sequence diagram displaying the typical interactions within a generic client. The time evolution of interactions is followed from top to bottom, while messages passed among objects are found in the horizontal dimension. The light yellow background of some lifelines indicate that these are threads.

Given the classes involved in the construction of this task-oriented, client-server paradigm for whole-body control, as well as the particular implementations for iCub, we present for the sake of clarity in Figure 1 an illustration of a typical server–client architecture with the underlying OCRA libraries used to build each component. This section proceeds with a time-based illustration of the interaction logic between the different objects of our system in the form of *sequence diagrams* (IEEE, 2009) as shown in Figures 2 and 3. Given the amount of classes in the package, it might be difficult to see the global interaction among them along with the intended architecture. The next two sections attempt to clear this out by showing the inner interactions of both client and server, independently and between them.

#### 4.1.3 iCub Server

Figure 2 depicts the sequence diagram for the ocra-icub-server. The user starts by executing the server from terminal issuing the command ocra-icub-server [options] (1).

The default options are specified in its initialization file ocra-icub-server.ini or hardcoded in the source code. After the execution of the server, an object of type ResourceFinder is created, which is responsible for the parsing of the former *options*. Right after, a yarp RFModule is created (3) and started (4), whose first task will be to configure the server (6), ask the ResourceFinder to find the desired type of controller (7), i.e., WOCRA or HOCRA, the solver to be used, i.e., QUADPROG or QPOASES, the XML file with the description of the tasks that the client will manipulate, etc. At this point, a yarpWholeBodyInterface object is created (8) and initialized. This class serves as an interface to the robot, and as such will allow us to set the control references obtained, as well as to obtain the state of the robot. Now the module is ready to create (12) and start (13) the main thread of the client.

Before entering the main loop of the thread, however, a couple of objects of interest are created. First, an object of type IcubControllerServer (14), which during initialization (16) will create the desired controller with its internal solver. At this phase, also communication ports are opened with standardized names that will be used by the cient for future connections. IcubControllerServer is then asked by the thread to update its internal model of the robot (17) and add the tasks specified by the user via XML (18). This process involves the creation (19) of an object of type TaskConstructionManager which will create one or multiple instances (20) of TaskBuilder, one per type of task found in the XML. These task objects will then get added to iCubControllerServer (21). Notice how the tasks are *living in the server*. The server will then ask the yarpWholeBodyInterface object to set the torque control mode on the robot (22) for it to accept torque references. The latter are computed every cycle of the Thread (24–27) by iCubControllerServer.

The server will be constantly controlling the robot to achieve default initial states of the specified tasks. As an example, if one task is of COM type, it controls the robot to keep it at its initial position, until a client connects to the server and tells it to do otherwise. Finally, if the user decides to stop the server (28), the sequence of object “destructions” is illustrated from (29) to (37).

#### 4.1.4 Generic Client

A client’s main goal is to connect to the server to provide reference trajectories to the tasks it hosts. Let us show through Figure 3 the main interactions within a client and the type of communication it establishes with the server.

As done previously on the server side, we are going to follow the sequence diagram in an orderly fashion. First, notice how before the user can start a client, they need to start the server. This is evident by the sequence number (2) next to example-client. Thus, having a server properly started, the client is launched and the first thing it does is to get model information of the robot through the class ModelInitializer. This is the first interaction between the client and the server (4-5), after which a local model of the robot is built (6). Once the client has access to the robot model, the main client thread is created (7). This is of type ControllerClient which is a Yarp RateThread. The creation of the thread is followed by a ClientCommunications object (8), which creates and connects local ports to the server for inter-process communications. Its role will become clearer later on. The client thread is passed to a ClientManager object (10) which will handle the life-cycle of the thread and its configuration (11–12). The module subsequently starts (18) the client thread, which afters initialization will spawn a couple of objects of interest.

Given the tasks contained in the XML file (taskSet) and fed to the server, the client will create one or more TaskConnection objects (18) for each of those tasks that are to be manipulated. Although not depicted in the diagram, for the sake of clarity, these objects will open control ports that are then connected to their corresponding tasks on the server side (19). It is through these objects that the client will be able to send task-specific messages to get or set their state.

As it is often the case, the user might want to create reference trajectories (of even different types) for all or some of the tasks. To this end one or more objects of type TrajectoryThread are created (20). These, at the same time, will internally create TaskConnection objects again to set the references to the tasks on the server (21). The client thread can then start the trajectory threads (23) and run in the background until it receives new references (25–29).

Now that the client has created task connections and trajectory threads, the client logic starts in the main thread (30–40). In this main loop, the client can:

- Get or set task-specific states through the TaskConnection objects (31–34).
- Add, remove or get tasks through the ClientCommunications object (35–38).
- Set references to tasks trajectories through the TrajectoryThread objects (39–40).

In order to stop the client, the user can send a SIGINT signal (ctrl + c) to kill the process and the sequence of “destructions” will be as in (43–53).

In Section 5.2, a link to a short tutorial can be found where it is explained how to launch a server and client.

#### 4.1.5 Client Generator

Because each new iCub controller client requires the same basic setup, a helper tool has been developed to automatically scaffold out the minimum required code for a new client. Invoking icub-client-generator [name-of-client] from the command line will produce a directory called name-of-client/, with all of the minimum client requirements and a complete CMake build. One then needs only to edit the name-of-client.cpp file and add control logic. Therefore, anyone can write an iCub client in just a few minutes.

## 5 Conclusion

The development of intelligent and autonomous robots entails many challenges, one of which is robust and flexible controllers. The overall goal of any control software should be to abstract the control of redundant robots, such as the iCub, to higher and higher levels of logic in order to facilitate the generation of complex overall behaviors—behaviors, which should ultimately render the robot useful. Whole-body control was born from these requirements and lays forth the design criteria for OCRA presented in Section 1. Through its various abstract and concrete classes, and server–client structure, OCRA attempts to provide a solution which meets these needs but also balances ease of use with flexibility. The design of OCRA allows users to interact with and customize the control problem at virtually any level from the real-time computation of joint torques to high-level controller clients. This wide array of usability means that OCRA is suitable for any user from control experts to control novices. We believe that this is an important step toward improving the usability of such software because the learning curve should be simple for those who only want a functioning controller, but the software should also be flexible enough to allow users to experiments with fundamental concepts.

At the low-level, this is accomplished by abstracting the various aspects of the control problem and providing concrete implementations for the most commonly reused concepts. Users interested in low-level control concepts can, therefore, experiment with customizing the abstract interface classes to their own needs, or simply construct novel controllers using the concrete class implementations. Higher-level usage on the other hand, is easy to get started with, thanks to the server–client architecture. If the robot has been properly interfaced with the OCRA controller server, then clients can be developed with little effort and most of all, no deep understanding of the internals of the server side. Various examples of the different manners in which one can interact with OCRA are presented in the Supplemental Data Section and validate the variety of ways OCRA can be used to study and develop autonomy.

Ultimately, OCRA should serve as the basis for increasingly complex logic, by robustly resolving progressively more complex layers of the control problem. The server–client architecture is just the beginning of this process and should be built upon by even high-levels of problem reasoning, to create greater and greater levels of robot autonomy.

## Author Contributions

GE, RL, and AH contributed to the development and integration of the proposed software framework. VP laid out the conceptual foundations of the main algorithms in this software. GE, RL, AH, and VP contributed to the writing of the associated paper, JE being the main contributor to the writing.

## Conflict of Interest Statement

The authors declare that the research was conducted in the absence of any commercial or financial relationships that could be construed as a potential conflict of interest.

## Footnotes

^1^http://www-list.cea.fr/en/.

^2^European Project Whole-body Compliant Dynamical Contacts in Cognitive Humanoids.

## Online Material

Website: https://ocra-recipes.github.io/web/.

OCRA Documentation: https://ocra-recipes.github.io/web/doxy-ocra-recipes/html/index.html.

OCRA iCub Documentation: https://ocra-recipes.github.io/web/doxy-ocra-wbi-plugins/html/index.html.

OCRA Source Code: https://github.com/ocra-recipes/ocra-recipes.

OCRA iCub Source Code: https://github.com/ocra-recipes/ocra-wbi-plugins.

Related publications: https://ocra-recipes.github.io/web/authors/.

Tutorials: https://ocra-recipes.github.io/web/icub/2016/11/26/using-ocra-with-icub.html.

## References

[B1] Bouyarmane K. Kheddar A. (2011). “Using a multi-objective controller to synthesize simulated humanoid robot motion with changing contact configurations,” in IEEE/RSJ International Conference on Intelligent Robots and Systems (IROS), 2011 (San Francisco, CA: IEEE), 4414–4419. 10.1109/IROS.2011.6094483

[B2] Escande A. Mansard N. Wieber P.-B. (2014). Hierarchical quadratic programming: fast online humanoid-robot motion generation. Int. J. Rob. Res. 33, 1006–1028. 10.1177/0278364914521306

[B3] IEEE. (2009). 1016-2009 – IEEE Standard for Information Technology–Systems Design–Software Design Descriptions (IEEE). 10.1109/IEEESTD.2009.5167255

[B4] Khatib O. (1986). Real-time obstacle avoidance for manipulators and mobile robots. Int. J. Rob. Res. 5, 90–98. 10.1177/027836498600500106

[B5] Khatib O. (1987). A unified approach for motion and force control of robot manipulators: the operational space formulation. IEEE J. Rob. Autom. 3, 43–53. 10.1109/JRA.1987.1087068

[B6] Liu M. Tan Y. Padois V. (2016). Generalized hierarchical control. Auton. Robots 40, 17–31. 10.1007/s10514-015-9436-1

[B7] Mansard N. Stasse O. Evrard P. Kheddar A. (2009). “A versatile generalized inverted kinematics implementation for collaborative working humanoid robots: the stack of tasks,” in International Conference on Advanced Robotics, 2009. ICAR 2009 (Munich: IEEE), 1–6.

[B8] Mistry M. Buchli J. Schaal S. (2010). “Inverse dynamics control of floating base systems using orthogonal decomposition,” in IEEE International Conference on Robotics and Automation (Anchorage, AK: IEEE), 3406–3412. 10.1109/ROBOT.2010.5509646

[B9] Nori F. Traversaro S. Eljaik J. Romano F. Del Prete A. Pucci D. (2015). iCub whole-body control through force regulation on rigid non-coplanar contacts. Front. Rob. AI. 2:6 10.3389/frobt.2015.00006

[B10] Padois V. (2016). Control and Design of Robots With Tasks and Constraints in Mind. Paris, France: Hdr, Université Pierre et Marie Curie (Paris 6).

[B11] Padois V. Fourquet J.-Y. Chiron P. (2007). Kinematic and dynamic model-based control of wheeled mobile manipulators: a unified framework for reactive approaches. Robotica 25, 157–173. 10.1017/S0263574707003360

[B12] Peters J. Mistry M. Udwadia F. Nakanishi J. Schaal S. (2008). A unifying framework for robot control with redundant dofs. Auton. Robots 24, 1–12. 10.1007/s10514-007-9051-x

[B13] Roboptim. (2016). C++ Library for Numerical Optimization for Robotics. Available at: http://roboptim.net/

[B14] Rocchi A. Hoffman E. M. Caldwell D. G. Tsagarakis N. G. (2015). “Opensot: a whole-body control library for the compliant humanoid robot coman,” in IEEE International Conference on Robotics and Automation (ICRA), 2015 (Seattle, WA: IEEE), 1093–1099. 10.1109/ICRA.2015.7140076

[B15] Saab L. Ramos O. E. Keith F. Mansard N. Soueres P. Fourquet J.-Y. (2013). Dynamic whole-body motion generation under rigid contacts and other unilateral constraints. IEEE Trans. Robot. 29, 346–362. 10.1109/TRO.2012.2234351

[B16] Salini J. (2012). Dynamic Control for the Task/Posture Coordination of Humanoids: Toward Synthesis of Complex Activities. Theses, Paris: Université Pierre et Marie Curie – Paris VI.

[B17] Salini J. Ivaldi S. Hak S. Padois V. (2013). ISIR Controller in the XDE Framework for the Control of Robots Based on LQP Solvers. Available at: http://chronos.isir.upmc.fr/salini/XDE-ISIRController/documentation/html/index.html

[B18] Salini J. Padois V. Bidaud P. (2011). “Synthesis of complex humanoid whole-body behavior: a focus on sequencing and tasks transitions,” in IEEE International Conference on Robotics and Automation (ICRA), 2011 (Shanghai: IEEE), 1283–1290. 10.1109/ICRA.2011.5980202

[B19] Sentis L. Khatib O. (2005). “Control of free-floating humanoid robots through task prioritization,” in Proceedings of the 2005 IEEE International Conference on Robotics and Automation, 2005. ICRA 2005 (Barcelona: IEEE), 1718–1723. 10.1109/ROBOT.2005.1570361

[B20] Sentis L. Khatib O. (2006). “A whole-body control framework for humanoids operating in human environments,” in Proceedings 2006 IEEE International Conference on, Robotics and Automation, 2006. ICRA 2006 (Orlando, FL: IEEE), 2641–2648. 10.1109/ROBOT.2006.1642100

